# The role of lncRNA-mediated pyroptosis in cardiovascular diseases

**DOI:** 10.3389/fcvm.2023.1217985

**Published:** 2023-06-16

**Authors:** Bo Hu, Wen Chen, Yancheng Zhong, Qinhui Tuo

**Affiliations:** Hunan Key Laboratory of Vascular Biology and Translational Medicine, Medical School, Hunan University of Chinese Medicine, Changsha, China

**Keywords:** cardiovascular disease (CVD), LncRNA – long noncoding RNA, gasdermins (GSDMs), pyroptosis, inflammatory

## Abstract

Cardiovascular disease (CVD) is the leading cause of death worldwide. Pyroptosis is a unique kind of programmed cell death that varies from apoptosis and necrosis morphologically, mechanistically, and pathophysiologically. Long non-coding RNAs (LncRNAs) are thought to be promising biomarkers and therapeutic targets for the diagnosis and treatment of a variety of diseases, including cardiovascular disease. Recent research has demonstrated that lncRNA-mediated pyroptosis has significance in CVD and that pyroptosis-related lncRNAs may be potential targets for the prevention and treatment of specific CVDs such as diabetic cardiomyopathy (DCM), atherosclerosis (AS), and myocardial infarction (MI). In this paper, we collected previous research on lncRNA-mediated pyroptosis and investigated its pathophysiological significance in several cardiovascular illnesses. Interestingly, certain cardiovascular disease models and therapeutic medications are also under the control of lncRNa-mediated pyroptosis regulation, which may aid in the identification of new diagnostic and therapy targets. The discovery of pyroptosis-related lncRNAs is critical for understanding the etiology of CVD and may lead to novel targets and strategies for prevention and therapy.

## 1. Introduction

In recent decades, the development of pyroptosis has brought a fresh notion for the pathophysiology of cardiovascular illnesses (1). Pyroptosis-related proteins gasdermins (GSDMs) generate porous membrane channels, and cell membrane rupture results in the release of inflammatory factors and more severe inflammatory responses (2–5). Long non-coding RNAs (LncRNAs) (6) are non-coding RNA molecules that contain more than 200 nucleotides in eukaryotes. These non-coding RNAs serve a variety of biological roles, including molecular scaffolding in the nucleus, variable splicing assistance, chromosomal shape regulation, translation regulation, and promoting or inhibiting messenger RNA (mRNA) breakdown in the cytoplasm (7). Several investigations have found that lncRNA-mediated pyroptosis is linked to the onset and progression of CVD (8, 9). However, the precise involvement of lncRNA-mediated pyroptosis in CVD is unknown. The function of lncRNA-mediated pyroptosis in CVD is discussed in this research (Table 1).

**Table 1:** LncRNAs-mediated pyroptosis with characterized functions in cardiac diseases.

LncRNA	Class	Targets	Species	LncRNA category	Effect of pyroptosis	Related Disease	Function
Kcnq1ot1	Antisense	CDKN1C	Human	Signal	−	Diabetic cardiomyopathy, acute myocardial damage, arrhythmia	Regulates pyroptosis and fibrosis of cardiac fibroblasts treated with high glucose (10)
KLF3-AS1	Antisense	Sirt1	Human, mouse	Sponge	+	Myocardial infarction	Reduce the pyroptosis of myocardial cells and the infarct size of myocardial tissue (11)
TUG1	Intergenic	XIAP	Mouse	Sponge	+	Coronary microembolization	Relieve lipopolysaccharide induced myocardial cell injury and pyrosis (12)
SOX2OT	Intronic	TLR4	Mouse	Sponge	-	Coronary microembolization	Regulation of NF-κB mediated pyroptosis of myocardial tissue (13)
FAF	Intergenic	PAK2	Mouse	Unkonw	+	Acute myocardial infarction	Reduce myocardial cell pyroptosis, improve cell vitality, reduce myocardial infarction area (14)
GAS5	Intergenic	AHR	zebrafish, mouse	Sponge	+	Atherosclerosis,Diabetic cardiomyopathy	Improve cardiac function and myocardial hypertrophy caused by activation of pyrotic inflammasome (15)
PVT1	Intronic	Unkonw	zebrafish, mouse	Sponge	−	Myocardial ischemia reperfusion	Ameliorating myocardial ischemia-reperfusion injury by inhibiting Gasdermin D-mediated pryoptosi of cardiomyocytes (16)
MIAT	Intergenic	miR-214-3p	Human	Decoy	−	Diabetic cardiomyopathy	Regulate the expression of inflammatory factors in diabetic cardiomyopathy mediated by pryoptosis (17)
TINCR	Intronic	YTHDF2	zebrafish, mouse	Sponge	−	Diabetic cardiomyopathy	Positively modulated NLRP3 by increasing the stability of NLRP3 mRNA (18)
ANRIL	Antiense	CDKN2A	Human	Scaffold	+	Myocardial infarction	Genetic risk factor for coronary artery disease (19)
Rian	Intergenic	CCND1	Human	Sponge	+	Myocardial ischemia reperfusion	Reduce myocardial pryoptosi and myocardial ischemia reperfusion injury (20)
HCG11	Intergenic	JAK1	Human, mouse	Sponge	−	Atherosclerosis	Alleviating pyroptosis induced by oxidative low density lipoprotein (ox-LDL) (21)
H19	Intergenic	SOX9	Human	Unkonw	+	Atherosclerosis	Alleviating pyroptosis induced by oxidative low density lipoprotein (ox-LDL) (22)
NEAT1	Intergenic	miR-22-3p	Mouse	Guide	−	Myocardial infarction	Promote the activation of inflammatory bodies in macrophages (23)

LncRNAs regulate cardiac development and diseases through various mechanisms.

## 2. Overview of pyroptosis

Pyroptosis, also known as inflammatory cell necrosis, is characterized by the body's programmed recognition of intracellular pathogens and defence, resulting in constant cell expansion until the cell membrane bursts, followed by the release of intracellular contents and pro-inflammatory mediators into the cell's exterior to activate a robust inflammatory response (2). Pyroptosis is a type of programmed cell death that occurs quicker than apoptosis and regulated by inflammatory protease activity. Inflammatory proteases are the caspases (cysteine-dependent aspartic specific proteases) family members. From the inception of the word “pyroptosis” to the finding of the caspase protein family implicated in pyroptosis inflammation, representing a significant advance in cell death research has been a long road.

Friedlander was the first to reveal that Anthrax lethal toxin (LT) may trigger cell death and fast release of cell contents in primary mouse macrophages in 1986 (24). Two related discoveries in 1989 and 1996 respecteively showed that inflammatory medium, interleukin-1-converting enzyme (ICE) could convert precursor IL-1 to adult IL-1 (25–27), and that invasive plasmid antigen B (ipaB) of Shigella flexion could directly bind to ICE and activate ICE enzymes in infected macrophages (28). This type of cell death was initially thought to be apoptosis because some of its features, such as caspase dependence, DNA damage, and nuclear aggregation, were similar to apoptosis. However, subsequent studies have found that this form of death is distinct from apoptosis - in 2001 proposed the term sclerosis to describe proinflammatory programmed cell death, which distinguishes between pyroptosis and apoptosis (a non-inflammatory program of cell death) (29). It was first suggested that inflammasomes could activate inflammatory cysteine proteases and process IL-1β in 2002 (30). As studies progressed, it was found that atypical caspase-11 could also induce cell death in host infection with Salmonella independent of caspase-1 (31). During pyroptosis, the N-terminal domain of cleaved Gasdermin D (GSDMD) can oligomerize in the cell membrane to form pores and induce cell membrane rupture (2, 3). This GSDMD pore formation is thought to be a critical factor in the early onset of pyroptosis, causing plasma membrane rupture and cell swelling.

## 3. Gasdermin and the mechanism of pyroptosis

**Figure 1:**
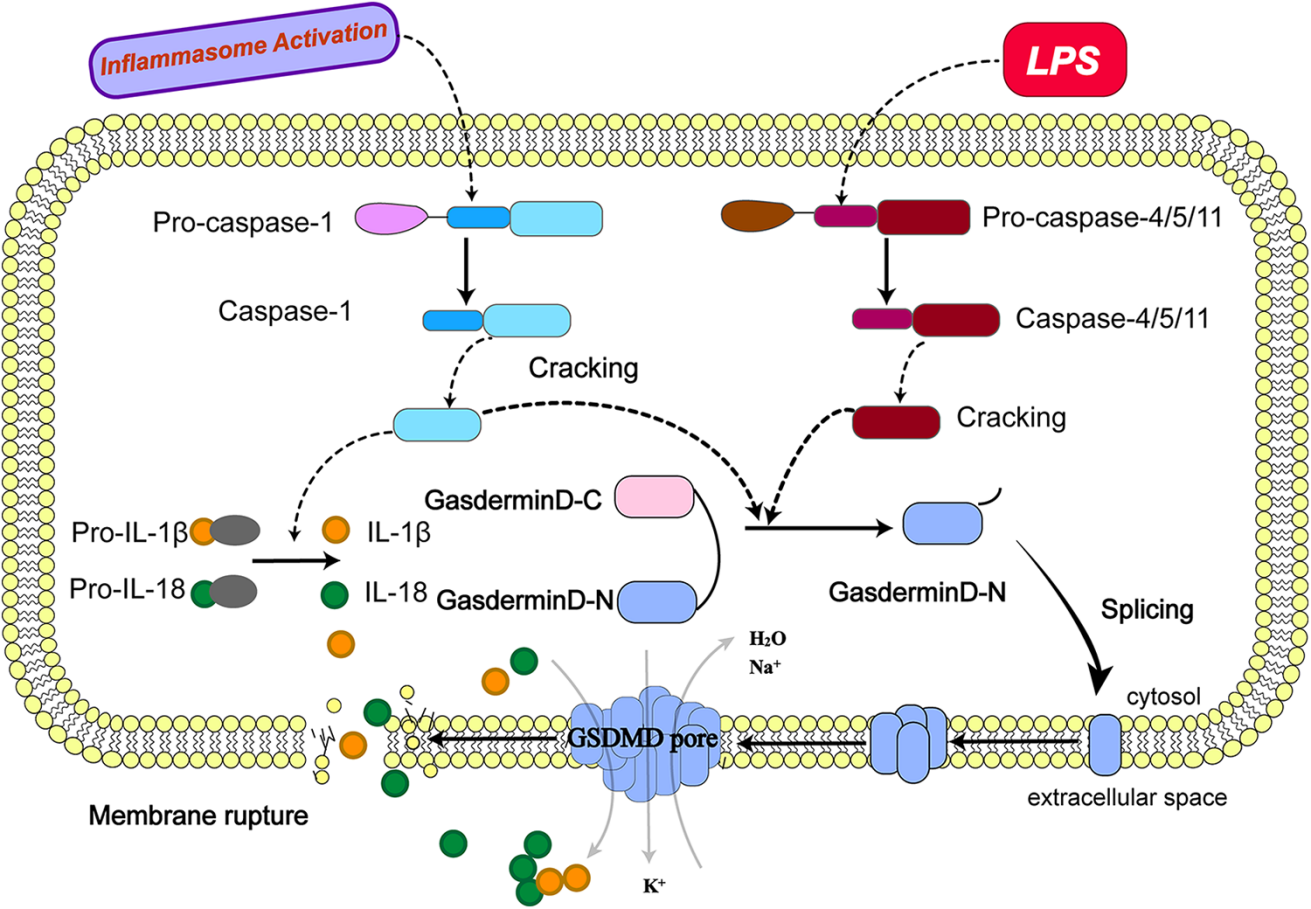
Molecular mechanism of pyroptosis. (The activation of pyroptosis can be divided into classical caspase-1 dependent pathways and non-classical caspase-1 independent pathways. The precursor caspase-1 protein complex is activated after recognizing the pathogen's stimulus signal. Activated caspase-1, cleaved GSDMD to form peptides containing the active domain of the nitrogen end of GSDMD, induced cell membrane perforation, cell rupture, release of contents, and cause inflammation; meanwhile, activated caspase-1 cleaves the precursors of IL-1β and IL-18 to form active IL-1β and IL-18, which are released into the extracellular space to recruit inflammatory cells to gather and amplify the inflammatory response. Bacterial lipopolysaccharides (LPS) bind to human caspase-4/5 and mouse caspase-11, mediating inflammation and causing cell necrosis. On the one hand, caspase-4/5 and 11 can be activated in response to the stimulation of LPS and other signals, the activated caspase-4/5 and 11 can cleave GSDMD and form peptides containing the active domain of the nitrogen end of GSDMD; On the other hand, they induce the activation of caspase-1, which cleaves the precursors of IL-1β and IL-18 to form active IL-1β and IL-18 and releases them outside the cell, recruiting inflammatory cells to gather and amplifying the inflammatory response.).

The GSDM family (32) contains six members, GSDMA, GSDMB, GSDMC, GSDMD, GSDME and DFNB59 (33), of which GSDMD and DFNB59 have received the most attention in the study of pyroptosis (34). GSDMD was considered as an executor of pyroptosis for the first time in 2015 (32). The N-terminal structural domains of all members of this family function to induce cellular pyroptosis-except for DFNB59. All GSDM members, such as GSDMD, consist of two conserved structural domains, amino-terminal and carboxy-terminal, linked by an intermediate flexible amino acid linker that contains the enzymatic cleavage site for highly conserved caspases. Its amino-terminal (N-terminal) structural domain has a molecular weight of 30 kDa can form a Gasdermin pore (35, 36), also known as a Pores-forming domain (PFD), while the carboxy-terminal (C-terminal) structural domain with a controlled molecular weight size of 20 kDa is also known as a Repressor domain (RD). RD can be involved in the control of PFD activity, both structural fields are involved in the induction of pyroptosis. The RD can be applied in possession of PFD activity, and both structural domains are interested in installing pyroptosis (37). When the amino acid linker between the two structural domains is cleaved, the RD is separated from the PFD (38). The released PFD is integrated into the cell membrane, where large aggregates of PFD monomers form 10∼15 nm diameter pores (39–41) (Figure 1). The essence of cell pyroptosis occurs due to the appearance of GSDMD pores on the cell membrane surface, causing a leaky plasma membrane to erupt into an inflammatory response. The open GSDMD pores break the normal permeability barrier of the plasma membrane. In terms of ion homeostasis, the presence of the cleavage pore allows the balance of the concentration gradient inside and outside the cell to be disrupted, resulting in sodium ions being drawn into the cell by the force of the concentration gradient and the electrical gradient, the most immediate damage when sodium ions flow into the cytoplasm is the entry of large amounts of water into the cytoplasm, resulting in an increase in cell volume; in terms of inflammatory factors, the pyroptosis process consists of the cleavage of IL-1β and IL-18 by Caspase-1, before the membrane The mature cytokines are produced before rupture and are present in the cytoplasm as cytoplasmic restriction proteins. The 10∼15 nm diameter gastrin pore is much larger than IL-1β (4.5 nm) and IL-18 (5.0 nm) (39) after pyroptosis activation to produce lytic pores, releasing them from cells before they are lysed, triggering the severe inflammatory response.

Nowadays, research on the molecular mechanisms that induce cell death by pyroptosis has focused on the caspase-1-dependent classical pathway and the caspase-1-independent non-classical pathway (42).

### 3.1. Caspase-1-dependent classical pathway

As mentioned earlier, upon recognition of stimulatory signals from pathogens such as bacteria and viruses, the intracellular pattern expression receptors activate the inflammatory vesicle junction protein caspase recruitment domain (ASC) to bind with pro-caspase-1 protein forming a protein complex (43) and facilitates the activation of caspase-1 (44, 45). The activated caspase-1, cleaves GSDMD to form a peptide containing the nitrogen-terminal active domain of GSDMD and induces cell membrane perforation and cell rupture, releasing the contents and causes an inflammatory response.The activated caspase-1 can also cleave the precursors of IL-1β and IL-18 to form active IL-1β and IL-18, which are released into the extracellular space and recruit inflammatory cells to aggregate and amplify the inflammatory response (46, 47).

### 3.2. Caspase-1-independent non-classical pathway

In contrast to the classical pathway, human caspase-4/5 and mouse caspase-11 can bind to bacterial lipopolysaccharides (LPS), mediating inflammation and leading to cellular necrosis (48, 49). Caspase-4/5 and 11 can also be activated in response to stimulation by signals such as bacteria. Activated caspase-4/5 and 11 cleave GSDMD and form peptides containing the nitrogen-terminal active domain of GSDMD (50, 51). GSDMD induce cell membrane perforation, cell rupture and release of contents, causing an inflammatory response. GSDMD induces activation of caspase-1and cleaves the precursors of IL-1β and IL-18, forming active IL-1β and IL-18, and releasing them extracellularly to recruit inflammatory cells to aggregate and amplify the inflammatory response. In addition, activated caspase-11 can induce cellular inflammatory reactions by inducing membrane pore formation in GSDMD cells and promoting K + efflux through the Pannexin-1/ATP/P2X7 pathway, mediating NLRP3/ASC/caspase-1 activation and promoting IL-1β maturation and release. Both of these activation pathways can cleave GSDMD to induce the formation of cell membrane gaps and also stimulate the activation of IL-1β and IL-18 to promote a more intense extracellular inflammatory response.

Another caspase-3-dependent pyroptosis pathway differing from caspase-1/4/5/11 has recently been identified, which induces GSDMD-dependent pyroptosis, in that caspase-3 induces cell pore formation by cleaving GSDME and promoting the recruitment of the GSDME-N structural domain to the cell membrane, leading to pyroptosis (39). The distribution and expression level of GSDME determines how caspase-3 activates cell death. When cells overexpress GSDME, activated caspase-3 induces cell pyroptosis, whereas, for cells with low GSDME expression, caspase-3 induces apoptosis after activation of caspase-3 induces apoptosis. This caspase-3-dependent mode of cell death is known as apoptosis-like pyroptosis (52).

To some extent, pyroptosis is a double-edged sword. On the one hand, moderate pyroptosis contributes to cellular homeostasis and can effectively prevent excessive cell proliferation, thereby protecting the host (53).On the other hand, high levels of pyroptosis can lead to inflammation, which is detrimental to the maintenance of homeostasis *in vivo*. There is growing evidence that pyroptosis may be an effective anti-microbial defence mechanism in the host during infection. Pyroptosis eliminates pathogenic bacteria by lysing infected cells and exposing pathogens to extracellular defences, such as by telling pathogens to neutrophils with solid anti-microbial activity. In addition, pyroptosis facilitates pathogen clearance by sensing an alarm signal from immune cells recruited to the site of infection. During pyroptosis, IL-1β (54) and IL-18 are released extracellularly as cell integrity is disrupted, promoting leukocyte infiltration and activation. The release of inflammatory mediators by cell lysis, including factors such as ATP (55), IL-1 and heat shock proteins, stimulates the production of pro-inflammatory cytokines by activating pattern recognition receptors, which contribute to the control and eventual resolution of microbial infections and their return to a homeostatic state (2, 56).

However, mutations in intracytoplasmic receptors [NOD-like receptors (NLR)] or the persistence of sterile inflammatory stimuli can lead to excessive pyroptosis (2), which can be detrimental to the host and, if left unchecked, can lead to disease (57, 58). Excessively pyroptosis cells increase the inflammatory mediators IL-1β, IL-18 and the alarm protein HMGB-1. HMGB-1 has been reported to cause severe sepsis and infectious shock (59). This can harm the host and, if left unchecked, can lead to disease.

## 4. The role of pyroptosis in cardiovascular disease

Pyroptosis causes structural changes in the cardiovascular system that result from pyroptosis-mediated pathological changes in vascular endothelial cells, vascular smooth muscle cells, monocytes and macrophages, such as injury, proliferation and fibrosis. For example, oxidized low-density lipoprotein (Ox-LDL)-induced endothelial cell pyroptosis can cause abnormal endothelial proliferation and vascular stenosis; oxidative stress (60), NLRP3 inflammatory vesicles and lncRNA-mediated pyroptosis can contribute to the formation of myocardial fibrosis (61); IL-1β and IL-18-mediated pyroptosis responses directly lead to myocardial hypertrophy, ventricular hypertrophy and myocardial ischemia and hypoxia in diabetic patients (62). The above-mentioned cellular activation by pyroptosis signalling pathways leads to the formation of myocardial fibrosis. The above-mentioned CVD caused by the activation of cell death by the pyroptosis signalling pathway are of increasing interest. Uncovering risk factors for the development of cell death in the pyroptosis pathway is of paramount importance.

The pyroptosis-regulated cell death is mainly regulated by various caspase-selective pathways, including caspase-1, in response to pathogenic and bacterial signalling stimuli, ultimately leading to the formation of GSDMD pore and the release of mature IL-1β, IL-18 and other inflammatory factors. This pathogen-activated pathway releases inflammatory factors and inflammatory bursts regulated by the expression of these proteins mediated by the transcriptional and translational involvement of miRNAs in their pathway networks (63). Long-stranded non-coding RNAs (LncRNAs), precursor molecules for small molecule RNAs (64), including miRNAs, have been shown to regulate the expression of downstream genes by interfering with the shearing of miRNAs. Here we focus on the role of lncRNA-mediated pyroptosis in the cardiovascular system.

## 5. Mechanism of lncRNA-mediated pyroptosis in causing cardiovascular diseases

Only <2% of the human genome is transcribed into protein-coding RNAs, and many RNA transcripts with limited or no protein-coding capacity are known as non-coding RNAs (65). LncRNAs are non-coding RNA molecules of more than 200 nucleotides in eukaryotes. In the regulation of transcription, lncRNAs can directly regulate transcription by forming R-loop structures to aggregate transcription factors or by interfering with Pol II transcription at target motifs, and can affect mRNA conversion in a variety of ways (66). In tissue and embryo development, lncRNAs are involved in the regulation of genomic imprinting, multipotential differentiation of stem cells, embryonic development, cardiac development, haematopoiesis and immune system, so measuring the expression of lncrnas in different partitions or disease states can help to understand their function, reveal their mechanism of action, or identify useful molecular markers (67). Based on the location on the genome, lncRNAs can be classified into sense lncRNAs, antisense lncRNAs, enhancer lncRNAs, intergenic lncRNAs, bidirectional lncRNAs and intronic lncRNAs based on their location concerning their neighbouring genes (68).

In recent years, various lncRNAs have regulated cardiac development and disease pathogenesis by mediating pyroptosis (68). LncRNAs located in the nucleus and regulate gene expression at the epigenetic level. In contrast, a small number of lncRNAs (15%) are present in the cytoplasm and are involved in regulating the translation process. In the nucleus, lncRNAs play a structural role in forming and maintaining subnuclear structural domains, together with various nuclear proteins. For example, lncRNA NEAT1 plays a crucial role in creating paraspeckles (a type of atomic body). Quinozod proposes a model in which lncRNAs establish nuclear structural domains with target genes and bind to proteins to form RNA-DNA-protein complexes. In brief, lncRNAs recruit proteins to modify chromatin, triggering conformational changes leading to transcriptional regulation. The model currently applies to all lncRNAs and is implemented through four different mechanisms:

Signalling lncRNAs (69) can respond to a variety of stimuli and thus precisely regulate gene expression, for example, the voltage-gated potassium channel subfamily KQT membrane 1 justice/antisense transcript 1 (kcnq1ot1) recruits histone methyltransferases (e.g., multicomb repressor replicon PRC2) to specific sites of action in chromatin and suppresses abnormal focal activity in cardiomyocytes by triggering chromatin methylation-induced transcriptional silencing (70). The molecular decoy lncRNA inhibits transcription in a manner that isolates regulatory factors, and several lncRNAs are known to have decoy mechanisms of action involved in pyroptosis-mediated cardiovascular diseases, such as myocardial infarct-associated transcription factors (MIAT) involved in selective shearing processes and anti-myocardial hypertrophy Heavy-stranded RNA transcript (MHRT) and lncRNA lung adenocarcinoma transcript 1 (MALAT1) have been reported to be involved in the regulation of endothelial cell function and vascular growth in the cardiovascular system (71). Guided lncRNAs (69, 71) are involved in the regulation of endothelial cell function and vascular growth by binding to ribonucleoproteins and directing them to specific targets, for example FENDRR can form a complex with PRC2 to silence the expression of target genes, or with TrxG/Mll proteins to activate gene expression; scaffold lncRNAs direct regulation in complexes with multiple transcriptional activators or repressors. CDKN2B-AS1 (also known as ANRIL) binds PRC1 and PRC2 to regulate chromatin methylation, leading to silencing of the INK4b-ARF- ANRIL has been identified as a genetic risk factor for coronary artery disease, and its expression levels correlate with left ventricular dysfunction after myocardial infarction (69, 72).

**Figure 2:**
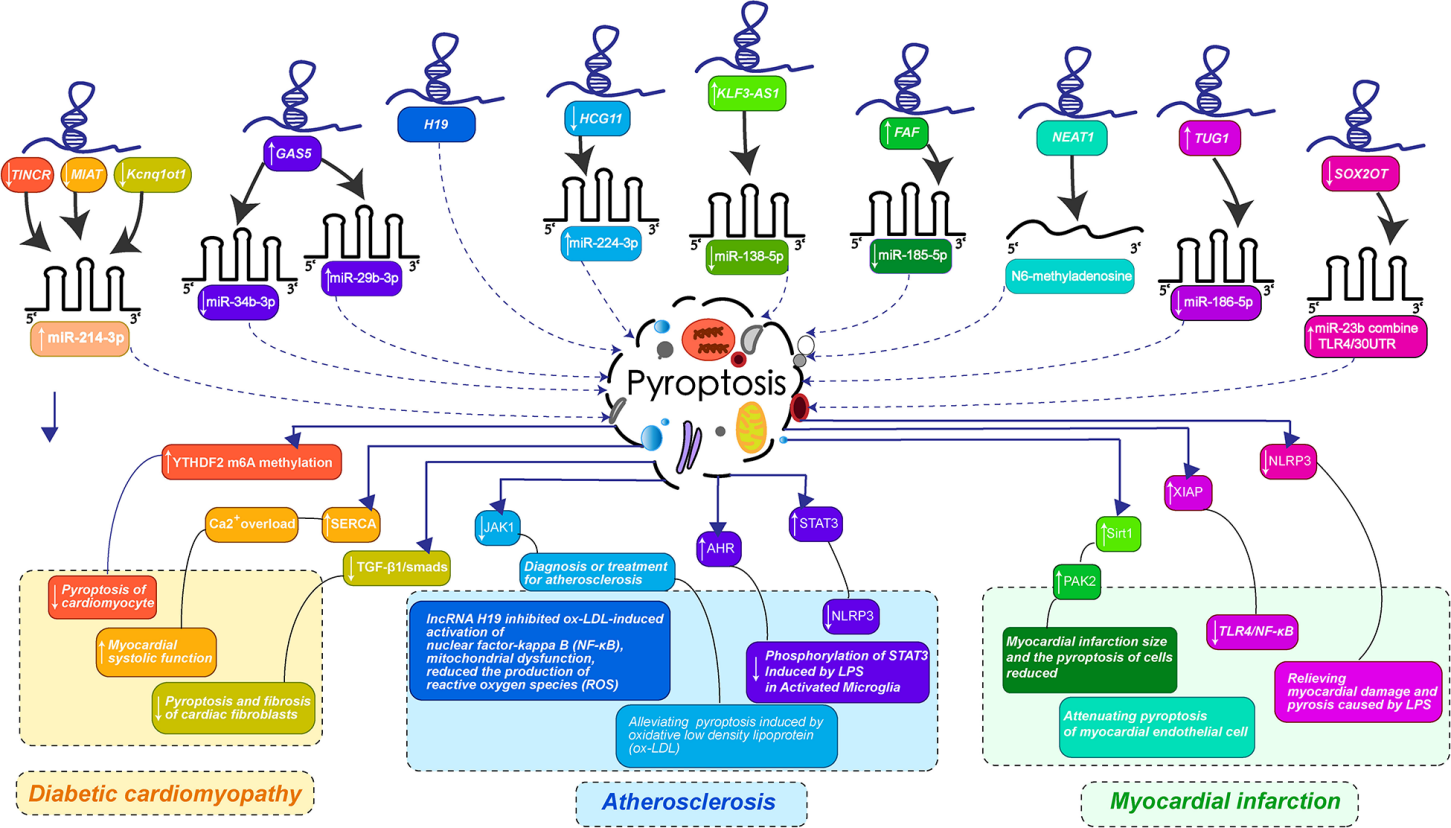
LncRNAs-mediated pyroptosis in cardiac development and diseases. (LncRNA-mediated pyroptosis regulates cardiovascular diseases through multiple mechanisms. The color of each lncRNA in the figure is the same as that corresponding to the color that guides the changes in the course of cardiovascular disease caused by miRNA-mediated pyroptosis. Diabetic cardiomyopathy was orange: the downregulation of TINCR, MIAT, and Kcnq1ot1 jointly promoted the expression of miR-214-3p, preventing pyroptosis-mediated myocardial fibrosis and enhancing myocardial systolic function. Atherosclerosis is bluish-purple: The overexpression of GAS5 inhibits miR-34b-3p, up-regulates miR-29b-3p, down-regulates HCG11 promoting the expression of miR-224-3p, H19 can directly act on pyroptosis, interfering with the activation of NF-κB by ox-LDL and reducing the release of inflammatory factor NLRP3. Myocardial infarction is turquoise and pink: KLF3-AS1 promoted the expression of miR-138-5p, FAF inhibited the expression of miR-185-5p, NEAT1 regulated the transformation of m6A, TUG1 inhibited the expression of miR-186-5p, and downregulation of SOX2OT promoted the binding of miR-23b and TLR4/30UTR. Thus, the myocardial infarction mediated by pyroptosis was improved and the myocardial cell injury induced by LPS was alleviated. The specific mechanism of lncRNA is shown in the figure.).

LncRNAs play a variety of roles in biological processes due to their different structures and mechanisms of action. Recent developmental experimental studies on lncRNAs have confirmed multiple mechanisms of regulating the process of cardiovascular diseases by mediating pyroptosis, including many complex diseases such as diabetic cardiomyopathy, atherosclerosis, myocardial infarction and other cardiovascular system diseases. The research focuses on exploring the mechanism of lncRNAs as a precursor of miRNA to interfere with the occurrence of pyroptosis of cardiomyocytes, the formation of fibrosis and lipid accumulation in the intima of blood vessels. These recent studies have greatly updated the existing understanding of lncRNA-pyroptosis-cardiovascular diseases, which can provide great help for clinical development to diagnose and treat cardiovascular diseases by utilizing the occurrence of pyroptosis. The mechanisms of lncRNA's effects on diabetic cardiomyopathy, atherosclerosis, myocardial infarction and other cardiovascular system diseases by mediating pyroptosis are summarized in Figure 2 and Table 1.

### 5.1. Diabetic cardiomyopathy

Diabetic cardiomyopathy (DCM) is a disease featured witn substantial cardiac lesion characterised by myocardial fibrosis and cardiac hypertrophy caused by chronic diabetes mellitus that occurs in the absence of hypertensive heart disease, coronary artery disease and heart valve disease (73). Several studies have shown that DCM is partially associated with pyroptosis, the most classic being myocardial fibrosis mediated by caspase-1 activation in a high-glucose environment: the lncRNA Kcnq1ot1, a signalling lncRNA, is highly expressed in the left ventricular tissue of diabetic mice, and a recent study by Fan Yang et al. showed that knockdown of Kcnq1ot1 by small interfering RNA reduced the expression of caspase-1 (10). The levels of NLRP3, caspase-1, IL-1β and GSDMD-N were significantly increased in the cardiac tissues of diabetic mice. However, their levels were significantly reduced by immunohistochemical analysis after kcnq1ot1 silencing (74). NLRP3, caspase-1 and IL-1β mRNA and protein expression levels were significantly elevated in diabetic (DM) mice and were reversed after kcnq1ot1-shRNA treatment. In addition, GSDMD-N protein expression levels were significantly elevated in diabetic mice and reversed after kcnq10t1 inhibition, and these effects were achieved through a complementary regulatory relationship between kcnq10t1 and miR-214-3p, caspase-1 and miR-214-3p (10). At the tissue level, kcnq1ot1 regulates caspase-1 expression in cardiac fibroblasts via miR-214-3p and thereby reduces irreversible myocardial fibrosis due to myocyte pyroptosis, as evidenced by the elevation induced by kcnq1ot1 silencing, leading to significant inhibition of miR-214-3p-targeted caspase-1 and its downstream inflammatory cytokine IL-1β is significantly inhibited (75). Previous studies have shown that IL-1β is closely associated with cardiac fibrosis and that prolonged exposure to IL-1β activates the TGF-β1 pathway, inducing the conversion of microvascular endothelial cells to myofibroblasts (76), leading to increased collagen synthesis, promotion of myocardial remodelling and increased interstitial myocardial fibrosis. lncRNA kcnq10t1 regulates pyroptosis in the diabetic heart possibly through kcnqot1/miR-214-3p/caspase-1/TGF-β1 pathway.

LncRNA Growth inhibitor-specific transcript (GAS5) has been reported to regulate various cardiac functions through pyroptosis (77), such as inhibition of cardiac hypertrophy, reduction of cardiac fibrosis and improvement of myocardial ischemia and hypoxia leading to cardiac dysfunction. Recent microarray analysis of lncRNA profiles in an *in vitro* DCM model (78) showed significant downregulation of GAS5 in cardiomyocytes. A study showed that GAS5 improved cardiac function and myocardial hypertrophy in DCM mice (15). GAS5, a competitive endogenous lncRNA, enhanced the aryl hydrocarbon receptor (AHR) expression by suppressing miR-34b-3p, thereby inhibiting NLRP3 inflammatory vesicle activation-mediated energy. miR-34b-3p upregulation in the DCM model significantly enhanced caspase-1 activity and levels of IL-1β and IL-18 in HL-1 cells, whereas GAS5 overexpression effectively abolished caspase-1 activity (79). LncRNA GAS5 regulates pyroptosis in the diabetic heart, possibly by targeting GAS5/miR-34b-3p/AHR to inhibit NLRP3 inflammatory vesicle activation in diabetic cardiomyopathy achieve.

In recent years, many lncRNAs have been reported to be involved in regulating diabetic cardiomyopathy by mediating pyroptosis. For example, MALAT1 expression is increased in the diabetic heart (80). Its knockdown improves cardiac contractile function and reduces inflammatory factors such as TNF-α, IL-6 and IL-1β in the diabetic myocardium, thus suggesting that MALAT1 may be involved in the inflammatory response to diabetic cardiomyopathy. Another study reported that MALAT1 upregulates inflammatory mediators TNF-α and IL-6 in high-glucose-treated endothelial cells by activating serum amyloid antigen. Large amounts of circulating lncRNA such as LIPCAR, SENCR and MIAT are valuable predictors of left ventricular diastolic function and remodelling in diabetic patients.

Many studies have revealed that several lncRNA, including MIAT and kcnq1ot1, are associated with DCM through inflammatory mechanisms. LncRNA myocardial infarction-associated transcript (MIAT) alleles, a lncRNA enriched in diabetic cardiomyocytes, act as competing endogenous RNAs (ceRNAs), forming a feedback loop with vascular endothelial growth factor. Furthermore, MIAT, CASP1 and IL-1b expression levels were upregulated and miR-214-3p expression levels were downregulated in serum of diabetic patients (81). In contrast, MIAT-silenced myocardial tissue of DM mice was associated with myocardial infarction compared to the regular group. Protein expression of caspase-1, IL-1b, IL-18, and GSDMD was significantly down-regulated and miR-214-3p expression was upregulated in MIAT-silenced myocardial tissue compared to the regular group, with the same results in both human and mouse genera (81, 82). Similarly, mRNA expression of caspase-1 and IL-1b was significantly reduced and miR-214-3p expression was elevated in mouse hearts following MIAT knockdown or caspase-1 inhibitor application, suggesting that MIAT may affect cellular pyroptosis in diabetic cardiomyopathy by targeting miR-214-3p to regulate the expression of caspase-1 and IL-1b process. It has been shown that MIAT is involved in both left ventricular diastolic function and myocardial remodelling in diabetic patients (83).

Pyroptosis-mediated inflammation in vascular endothelial cells and cardiomyocytes is essential in the late complications of cardiac dysfunction in diabetic patients. LncRNAs can regulate the development of diabetic cardiomyopathy by mediating the activation of pyroptosis-associated factors and affecting ventricular remodelling through multiple pathways that interfere with the integrity of cardiomyocytes and vascular endothelial cells, giving us many hints and motivation for research. The causal relationship and consequences of pyroptosis and chronic inflamation in cadiac myppathy are still to be further studied.

### 5.2. Atherosclerosis

CVD caused by atherosclerosis (AS) is the major cause of mortality in the global population (84). Pyroptosis as a form of inflammation-mediated cell death has become the focus of research to advance the pathological process of atherosclerosis (60, 85). Endothelial cell dysfunction is the earliest link in the development of atherosclerosis. Therefore, maintaining endothelial cells' integrity and normal function is essential to prevent and treat atherosclerosis (86).

Research on the role of lncRNAs in the pathology of atherosclerosis is still in its infancy. Several lncRNAs have been identified as being involved in the development of atherosclerosis (87). Atorvastatin is commonly used to control abnormal lipid metabolism in patients with atherosclerosis. Wu et al. demonstrated that atorvastatin can be expressed at both mRNA and protein levels by upregulating the expression of Nexilin F-actin binding protein antisense RNA1 (lncRNA NEXN-as1) and NEXN Reduction of NLRP3, caspase-1, GSDMD, IL-1β and IL-18 to inhibit pyroptosis (87). In RT-PCR analysis of samples from the clinic, RNA levels of NEXN and NEXN- as1 were lower in patients' atherosclerotic plaques than in healthy intimal tissue; their expression levels would be lower in advanced atherosclerotic plaque tissue. Recent study demonstrated that overexpression of NEXN-as1 inhibited NF-κB activity and downregulated endothelial cell adhesion molecules and inflammatory factors (87). These inhibitory effects of NEXN-as1 were abolished by knocking down NEXN: artificial knockdown of NEXN in Apoe knockout mice promoted atherogenesis, increased macrophage abundance in atherosclerotic lesions, and increased expression of adhesion molecules and inflammatory cytokines, whereas increased NEXN expression prevented atherosclerosis (88). In contrast, NEXN-as1 can protect against the progression of atherosclerosis by interacting with the chromatin remodeler BAZ1A and upregulating its homologous protein-encoding gene NEXN, thereby inhibiting endothelial cell activation and monocyte recruitment, ultimately providing protection against atherosclerosis progression (89). In addition, NEXN- as1 and NEXN may promote plaque stabilization by inhibiting adhesion molecules and extracellular matrix-degrading enzymes and reducing macrophage abundance and lipid content, hallmarks of vulnerable atherosclerotic plaques.

The expression of NLRP3 is increased in the aorta of patients with coronary artery disease, and its expression correlates with the severity of coronary artery disease (90). Various lncRNAs are involved in NLRP3 activation during pyroptosis, for example, lncRNA MALAT1 associated with NLRP, with low mustard acid reducing NLRP3 gene expression in macrophages by decreasing lncRNA MALAT1, and lncRNA MALAT1 promoting NLRP3 gene expression in myocardial ischemia-reperfusion injury (91–93).

In addition, recent studies suggest that the lncRNA-miRNA-mRNA regulatory network may play a vital role in the cellular pyroptosis process (8, 94). LncRNA MALAT1 may use this network to regulate NLRP3 expression by adsorbing endothelial cell miR-22 through a competitive inside-supporting RNA mechanism. By observing human umbilical vein endothelial cells transfected with miR-22 in a high glucose culture environment, the expression level of miR-22 decreased significantly after high glucose stimulation, while the level of lncRNA MALAT1 in endothelial cells changed in contrast to miR-22; overexpression of miR-22 could attenuate MALAT1's pyroptosis of endothelial cells induced by high glucose, suggesting that MALAT1/miR-22/NLRP3 regulatory pathway is involved in high-glucose-induced endothelial cell pyroptosis (95).

The involvement of the lncRNA-miRNA-mRNA regulatory network in mediating pyroptosis-induced endothelial cell damage and hence atherosclerosis applies to many existing studies: melatonin reduced ox-LDL-induced pyroptosis in human aortic endothelial cells by decreasing the lncRNA MEG3, and the possible molecular mechanism was that the adsorption of miR-223 by MEG3 unlocked the target gene NLRP3. Han et al. found (63) that low doses of erucic acid reduced the lncRNA MALAT1, thereby reducing macrophage pyroptosis in diabetic atherosclerotic rats, probably through the MALAT1/miR-23c/ELAYL1 pathway. LncRNAs may act as significant regulators of pyroptosis in atherosclerotic heart disease and are potential therapeutic targets, but further studies are needed to demonstrate their utility as biomarkers.

### 5.3. Myocardial infarction

Myocardial infarction (MI) is a severe coronary artery-related disease caused primarily by ruptured atherosclerosis or an imbalance in the supply and demand of oxygen to the heart muscle (96). When atherosclerosis ruptures, the released plaque collects platelets and causes occlusion of the coronary arteries, leading to ischaemic necrosis of the myocardium (97). Like atherosclerosis, pyroptosis is involved in cardiomyocyte injury by mediating the inflammatory response, and the pyroptosis pathway is also a potential therapeutic target for cardiovascular disease (98). It is considerable evidence that lncRNAs act as competing ceRNAs in myocardial infarction to regulate cellular biological activity in a manner that mediates the processes of the pyroptosis network.

Many recent bioinformatic predictions of the involvement of lncRNAs in mediating the pyroptosis pathway in regulating myocardial infarction have been confirmed (98). For example, the lncRNA KLF3-AS1/miR-138-5p/Sirt1 in exosomes from human mesenchymal stem cells (hMSCs) is involved in the protective effect on hypoxic cardiomyocytes (11). The myocardial infarction area was significantly reduced in rats with myocardial infarction injected with KLF3-AS1 exosome, and the expressions of NLRP3, caspase-1, GSDMD, IL-1β and IL-18 in KLF3-AS1-overexpressed myocardial infarction mice were also significantly decreased. The term of KLF3-AS1 and Sirt1 was upregulated after transfection with miR-138-5p inhibitor (99). Transfection with Sirt inhibitor reversed the protective effect of KLF3-AS1 on hypoxic cardiomyocytes, suggesting that lncRNA KLF3-AS1 acts as a competitive endogenous RNA by inhibiting miR-138-5p from regulating the expression of Sirt1 and thus inhibiting the activation of pyroptosis factor of the lncRNA KLF3-AS1, as a ceRNA, inhibits the activation of Sirt1 and thus protects cardiomyocytes (11, 100). Another study found that Sirt1 was downregulated in myocardial infarction and that Sirt1 overexpression effectively ameliorated myocardial injury caused by myocardial infarction. Data from vascular endothelial cells showed that Sirt1 inhibited the expression of NLRP3 and ASC in addition to the maturation of caspase-1 and the inflammatory cytokine IL-1β, suggesting that Sirt1 has an inhibitory effect on the activation of NLRP3 inflammatory vesicles and that Sirt1 has a protective effect on vascular endothelial cells.

Another bioinformatically predicted and validated pathway is the lncRNA FAF/miR-185-5p/PAK2 axis that mediates cardiomyocyte pyroptosis to ameliorate acute myocardial infarction. lncRNA FGF9 associated factor (FAF) is a recently identified pathway downregulated in infarcted myocardium and inhibits cardiomyocyte pyroptosis through transcriptional regulation of FGF9. Li et al. also demonstrated that the downregulation of miR-185-5p expression contributed to endothelial neovascularisation, which further reduced the decline in cardiac function in mice after infarction (101). Overexpression of the lncRNA FAF in the myocardium improved the pathological ultrastructural changes associated with acute myocardial infarction, such as myofilament lysis, loss of plasma membrane integrity, mitochondrial swelling and loss of cristae (102). Pathological changes. In addition, overexpression of myocardial lncRNA FAF in the rat myocardial ischemia/hypoxia model protected the membrane integrity of hypoxia/ischemia-induced focal cell death better than the control group. Recent report indicated that rat myocardial cells under hypoxia/ischemia induction showed significant cytosolic rounding, bubbles on the cell surface with bulging masses, and myocardial cells in the hypoxia and FAF overexpression group showed significantly reduced morphological differences (14). It has also been shown that modulation of miR-185-5p and PAK2 can exert cardioprotective effects. Furthermore, PAK2 expression was downregulated in hypoxic and ischemic cardiomyocytes. That overexpression of lncRNA FAF upregulated PAK2 protein levels under hypoxic conditions, reversed under transfected miR-185-5p co-culture conditions. Transfection with miR-185-5p inhibitor helped to reduce cellular pyroptosis, and in contrast, miR-185-5p mimicked the increased cellular pyroptosis under hypoxic ischemia. It is reasonable to believe that lncRNA FAF promotes the release of PAK2 by inhibiting the miR-185-5p pyroptosis pathway, which attenuates the cellular pyroptosis response and reduces the size of myocardial infarction.

In addition, pyroptosis-induced inferior coronary micro thrombosis is an essential pathogenesis of myocardial infarction. Coronary microemboli induce inflammatory responses and oxidative stress in the body in additiong to its blockage of blood stream. Microemboli leading to a collective burst of inflammatory reactions have recently been found to be mediated by various lncRNAs. LncRNA Sox2 overlapping transcripts (Sox2OT), a long-stranded non-coding RNA localized to the human chromosome 3q26.3 (Chr3q26.3) locus, a long-stranded non-coding RNA with an intron region containing the Sox2 gene, which is one of the primary regulators of pluripotency (13). Sox2OT was previously reported to enhance cardiac dysfunction by promoting re-releasing of reactive oxygen species (ROS) in mice with septic cardiomyopathy (103). Recently, the ability of Sox2OT to act as a competitive ceRNA in inducing various pyroptosis pathways has been increasingly demonstrated. In a recent survey, Xuan et al. concluded that silencing Sox2 downregulates NF-κB expression in cardiomyocytes, an effect that may be mediated by the miR-23b/TLR4/LPS pyroptosis pathway (13). Pyroptosis is a caspase-1-dependent form of programmed cell death inflammation. NF-κB upregulates pyroptosis-associated inflammasome NLRP3 levels, activates caspase-1 activity and promotes GSDMD expression, promoting the inflammatory response and inducing pyroptosis. In the rat infarct model, silencing of Sox2OT decreased NF-κb p65 phosphorylation and downregulated NLRP3, GSDMD and caspase-1 protein levels compared to the average infarct model, whereas overexpression of Sox2OT had the opposite result. RISC is a ribonucleoprotein complex containing proteins such as Argonaute (Ago) that binds mature miRNAs, mediates post-transcriptional gene silencing or mRNA target gene degradation, and is involved in the regulation of gene expression. The detection of lncRNA Sox2OT and miR-23b at the exact location and highly enriched using Ago magnetic antibody beads indicates that Sox2OT and miR-23b are in the same Ago complex. It was speculated that Sox2OT and miR-23b were in the same Ago difficult, the miR-23b level was increased, and the expression of TLR4 and NF-κb was significantly down-regulated in the silenced Sox2OT group. These results indicate that Sox2OT regulates TLR4 and mediates TLR4/NF-κb signalling pathway as a ceRNA of miR-23b. The effect of Sox2OT on miR-23b/TLR4/NF-κb may guide a novel therapeutic course for coronary embolism-induced acute heart attack. Therapeutic pathway for acute heart attacks caused by coronary embolism. More on the effect of lncRNAs on infarction lies in imaging ventricular remodelling, e.g., myocardial infarction-associated transcript 1 (Mirt1) expression correlates with the characterization of genes related to left ventricular remodelling (104); GAS5 overexpression improves new function and ventricular hypertrophy in a mouse model of myocardial infarction, all of which are of great research value.

In the study of lncRNAs mediating the development of acute myocardial infarction, many lncRNAs were found to be differentially regulated. Whether the regulatory effects of these lncRNAs, taken together, contribute to attenuating myocardial cell injury and ventricular remodelling by pyroptosis requires further validation.

## 6. Conclusion and prospects

Recent research on lncRNA-mediated pyroptosis has shed light on the processes behind the onset and progression of cardiovascular diseases. The suggested methods of lncRNA controlling pyroptosis by interfering with miRNA expression, in particular, have filled several major gaps in the notion of miRNA's function in cardiovascular disorders. An increasing number of biological research on the function of lncRNA-mediated pyroptosis in cardiovascular illnesses have shown that lncRNA-mediated pyroptosis will play an important role in the future screening, diagnosis, and treatment of cardiovascular disorders.

The findings of the study on lncRNA-mediated pyroptosis in the cardiovascular system have significant therapeutic implications. LncRNA GAS5, as previously noted, can enhance heart function and even cure signs of myocardial hypertrophy in DCM mice by suppressing NLRP3 activation. In diabetic cardiomyopathy patients, lncRNA can be employed as a key predictor of ventricular remodeling. In recent clinical trials, atorvastatin has been shown to reduce the occurrence of pyroptosis in patients with atherosclerosis by up-regulating the lncRNA NEXN-AS1, and the NEXN-AS1 RNA level in vascular intimal plaques in patients with atherosclerosis is also significantly lower than in healthy intimal tissues.

Although lncRNA-mediated pyroptosis are still limited to molecular biology and disease model experiments, the regulatory role of lncRNA-mediated pyroptosis in cardiovascular diseases, as well as its participation in the pathophysiological process of the cardiovascular system reflected by lncRNA, are bound to be applied and reflected in future clinical studies. The scene will be set for lncRNA-mediated pyroptosis to shine through early screening of high-risk groups for cardiovascular illnesses, as well as diagnosis and treatment of patients.

There are certain limitations in the research on the involvement of lncRNA-mediated pyroptosis in cardiovascular disorders that must be addressed. Pyroptosis is a necessary programmed death process that mediates inflammatory burst and cell death in cardiovascular diseases, and the wide range of transcript properties of lncRNAs and regulatory miRNAs is bound to shine through in the activating regulatory function of pyroptosis. The lncRNAs may be more broadly conserved across species, making determining their exact roles harder. Furthermore, translating discoveries from mice models to humans is still a considerable challenge. Because lncRNAs are more conserved at the functional and structural (secondary structure) levels than at the sequence level, lncRNA-mediated pyroptosis in cardiovascular diseases has only shown the association of lncRNAs with disease processes, with a few of these findings coming from original studies on tissue samples, but the majority still coming from bioinformatic engineering of publicly available databases such as Ensembl, GenBank, or the UCSC Genome Browser. The breadth of these discoveries is related to a wide spectrum of cardiovascular disorders, including coronary atherosclerotic heart disease, acute myocardial infarction, arrhythmias, diabetic heart disease, chronic heart failure, and congenital heart disease.

The following questions have dominated the original research on lncRNA-mediated pyroptosis control of cardiovascular disorders in the last 5 years: Is there any lncRNA-mediated focused death pathway interoperability? What signals initiate lncRNA transduction, and what controls them? What are the regulatory differences between numerous differentially expressed lncRNAs in the same disease? All of these fundamental concerns are being investigated in the context of lncRNA/miRNA/mRNA, a process that could take decades or perhaps centuries. Finally, the link between pyroptosis and other cell death procedural pathways (such as apoptosis, necroptosis, cuproptosis, and feroptosis) and their combined involvement in cell death processes in cardiovascular disorders is worth exploring in depth. We anticipate that their special networking mechanisms will have an enormous effect on clinical diagnosis and treatment efforts in the future.
